# The Association Between Caffeine Consumption and Academic Success in Makkah Region, Saudi Arabia

**DOI:** 10.7759/cureus.57975

**Published:** 2024-04-10

**Authors:** Maryam Dahlawi, Yasser B Hennawi, Mohammad Baharith, Muhjah Almurakshi, Ahdab Bawashkhah, Saif Dahlawi, Shahad B Alosaimi, Faisal S Alnahdi, Turki Talal Alessa, Omar Althobity, Majed Obaid

**Affiliations:** 1 Faculty of Medicine, Umm Al-Qura University, Makkah, SAU

**Keywords:** grade point average (gpa), saudi arabia, academic performance, caffeinated beverages, medical students

## Abstract

Background: The consumption of caffeinated beverages has increased significantly, particularly, among young adults. They use caffeinated drinks for a variety of reasons. The most popular reason is to enhance mental alertness by improving brain function, wakefulness, and productivity. The high prevalence rate of caffeinated drinks among young adults may affect their academic performance level.

Methodology: A descriptive cross-sectional study based on an electronic questionnaire via Google Forms, conducted in February 2022 after the biomedical ethics committee obtained the ethical approval at Umm Al-Qura University (UQU), College of Medicine, Makkah, Saudi Arabia, the sample size was 593 medical students in Makkah region.

Results: A total number of 593 medical students participated in this study, most of the medical students who participated (47.20%) had average GPA of 85%-95%. The largest number of medical students (45.30%) consume only one cup of caffeinated beverages per day. We reported no association between caffeinated beverages consumption and academic performance.

Conclusion: Our study demonstrates that caffeinated beverages are a popular practice among medical college students. Majority of the medical students in Makkah region consume coffee as the most popular drink, while energy drinks are considered to be the least consumed drink, but energy drinks are easily affordable and available. Therefore, primary prevention of excessive consumption of caffeine is essential.

## Introduction

Over the past two decades, the popularity of caffeinated beverages has increased dramatically, and their consumption has become a widespread phenomenon. Caffeinated drinks are any beverages that contains a high amount of caffeine, a stimulant popular in most developed countries. Despite widespread reports of negative side effects, the usage of caffeinated beverages has surged among college students, as they consume it to aid in completing their work. However, little is known regarding the link between caffeinated drink consumption and academic achievement [1]. Coffee and tea are the most popular natural caffeinated beverages. Additionally, soft drinks such as cola and energy drinks are also considered stimulant products [2]. Energy drinks typically contain higher concentrations of amino acids and herbals than those naturally found in food and plants. The energetic effect may be enhanced when caffeine is added [3-5].

People of all ages frequently consume caffeinated drinks. Previous studies have reported a significant increase in the prevalence of caffeinated beverage consumption, particularly among young adults [6,7]. In the United States, Malinauskas et al. found that approximately 51% of college students consume caffeinated beverages [8]. A similar pattern is observed in other developed countries, including Australia, Canada, and Italy [7,9].

Likewise, in the Middle East and North Africa (MENA) region, Ghozayel et al. observed a significant increase in caffeinated beverage consumption among young adults. Caffeinated beverages are widely consumed in MENA countries, raising serious concerns regarding potential health and safety consequences [4].

People use caffeinated drinks for a variety of reasons. One reason is to enhance mental alertness by improving brain function, wakefulness, and productivity [2]. Energy drinks are commonly used by tired individuals to help them stay focused, but this may lead to a decrease in sleep quality [4]. Surgeons specifically may use caffeinated drinks to decrease fatigue levels. Another reason is that caffeine may assist athletes in recovering faster from exercise and improve their physical performance [5]. However, it is also associated with serious health problems, such as increased heart rate, blood pressure, and heart rhythm disturbances [6].

Mahoney et al. investigated the reasons behind the use of caffeinated drinks among college students. The authors found that students commonly reported the following reasons: to combat sleep, enjoy the delicious taste, benefit from the social aspects of consumption, improve performance, increase energy, enhance mood, and alleviate stress [7].

It is suggested that due to their high prevalence among young adults, caffeinated drinks may affect academic performance levels. Therefore, this study aims to determine the frequency of caffeine consumption and its effect on academic performance among medical students in Makkah, Saudi Arabia.

## Materials and methods

This is a descriptive cross-sectional study based on an electronic questionnaire using Google Forms, conducted in February 2022 after obtaining ethical approval from the Biomedical Ethics Committee at Umm Al-Qura University (UQU), College of Medicine, Makkah, Saudi Arabia, with Approval No (HAPO-02-K-012-2022-02-958). The sample size was calculated using the Raosoft calculator based on the population size of medical students in the Makkah region, which was 7,200. The minimum sample size required to achieve a precision of 5% with a 95% CI was 365. The final sample size was 593. The survey was adapted from a previously validated questionnaire used in a published study [9]. It was publicized to medical students via social media platforms. Male and female students from the second medical year through internship year at the following medical colleges were included: UQU, King Abdulaziz University, King Saud University, Jeddah University, Ibn Sina University, Fakeeh College for Medical Science, and Taif University. Students who refused to participate were excluded. The questionnaire consisted of two sections. The first section collected demographic information such as age, gender, academic year, physical activity, BMI, smoking, type of caffeinated beverages consumed, frequency of consumption, and grade point average (GPA) to measure academic achievement. As it is not standardized across all included universities, there were two GPA scales used in this study: a GPA out of 4 and a GPA out of 5. Therefore, these variables were recorded using a percentage grade category, and divided into three categories as follows: <85%, 85%-95%, and >95%. These percentage categories reflect the following GPAs: <3.50/4.50, 3.50-3.75/4.50-4.75, and >3.75/4.75, respectively. Participants self-reported their overall GPA by selecting the percentage grade category that best reflected their GPA. The second part of the questionnaire assessed students' knowledge and perception of caffeine consumption through yes or no questions. The first author's email address was provided for any questions or issues, and all students provided informed consent.

Statistical analysis

The obtained data were initially gathered in an Excel sheet to be checked. Afterward, we used IBM SPSS for Windows version 23.0 (IBMCorp, USA) for data analysis. As our continuous variables were normally distributed, we reported them as mean ± standard deviation. Categorical variables are reported as frequencies and percentages. And significance utilizes the Chi-square test, with a <0.05 p-value to be considered statistically significant. We conducted a multinomial logistic regression model to explore the relationships between frequency of caffeinated beverage consumption and GPA. We did it by considering a GPA of <85% as the reference category, set at 95% CI, with p-values of <0.05 deemed statistically significant. 

## Results

Descriptive characteristics of participants

Table 1 presents the demographic characteristics of the medical students who participated in this study. A total of 593 medical students took part. 369 (62.2%) were female and 224 (37.8%) were male. Most participants, 348 (58.7%), fell within the age group of 21-23 years old. In terms of BMI scale, 299 (50.4%) of the participants had a normal BMI, 128 (21.6%) were overweight, 67 (11.3%) were underweight, 60 (10.1%) were obese, and 39 (6.6%) were extremely obese. Most participants, 254 (42.8%), had a sedentary lifestyle, followed by 156 (26.3%) with a low activity level. Among the participants, 69 (11.6%) were smokers, with 30 (46.9%) of them having a smoking duration of 1-3 years and 25 (39.2%) of 4-6 years. Most of the smokers, 24 (37.5%), consumed less than 10 cigarettes per day. Regarding income, most students, 431 (72.7%), had a monthly income of less than 1,500 SR. UQU had the highest number of participants, with 280 (47.2%), followed by Taif University, with 96 (16.2%). There was a variation in academic years, with 108 (18.2%) in the second year, 140 (23.6%) in the third year, 97 (16.4%) in the fourth year, 127 (21.4%) in the fifth year, 72 (12.1%) in the sixth year, and 49 (8.3%) as interns. 

**Table 1:** Demographic characteristics of medical students UQU: Umm Al-Qura University

Variables	n	%
Gender		
Male	224	37.8%
Female	369	62.2%
Age		
18-20 years old	146	24.6%
21-23 years old	348	58.7%
24-26 years old	99	16.7%
BMI		
<18.5 (underweight)	67	11.3%
18.5-24.9 (normal)	299	50.4%
25.0-29.9 (overweight)	128	21.6%
30.0-34.9 (obese)	60	10.1%
>35 (extremely obese)	39	6.6%
Physical activity		
Sedentary lifestyle (<5,000 steps per day)	254	42.8%
Low active (5,000-7,499 steps per day)	156	26.3%
Somewhat active (7,500-9,999 steps per day)	117	19.7%
Active (>10,000 steps per day)	51	8.6%
Highly active (>12,500 steps per day)	15	2.5%
Smoking		
Yes	69	11.6%
No	524	88.4%
Duration of smoking		
1-3 years	30	46.9%
4-6 years	25	39.2%
>6 years	9	14.1%
Number of cigarettes		
>10 cigarettes per day	24	37.5%
10-15 cigarettes per day	13	20.4%
>15 cigarettes per day	10	15.7%
Electronic cigarettes	17	26.6%
Total personal income per month		
Less than 1,500 SR	431	72.7%
1,500-2,000 SR	84	14.2%
More than 2,000 SR	78	13.2%
University		
UQU	280	47.2%
King Abdulaziz University	30	5.1%
King Saud University	39	6.6%
Jeddah University	43	7.3%
Ibn Sina University	53	8.9%
Fakeeh College for Medical Science	7	1.2%
Batterjee Medical College	45	7.6%
Taif University	96	16.2%
Academic year in medical school		
2^nd^ year	108	18.2%
3^rd^ year	140	23.6%
4^th^ year	97	16.4%
5^th^ year	127	21.4%
6^th^ year	72	12.1%
Intern	49	8.3%

**Figure 1:**
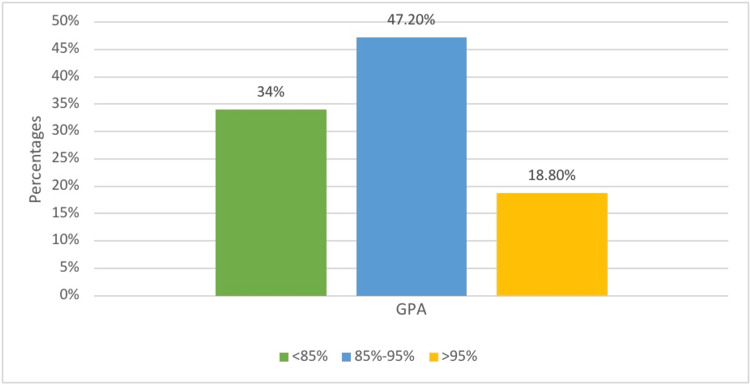
Academic performance of medical students by GPA percentages GPA: Grade point average

**Figure 2:**
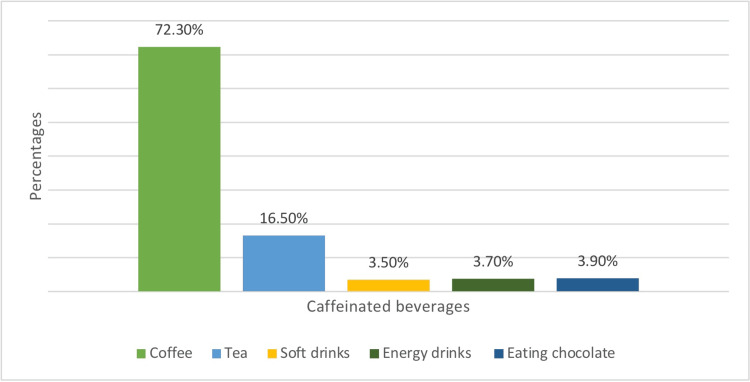
Caffeinated beverages consumed by medical students

**Figure 3:**
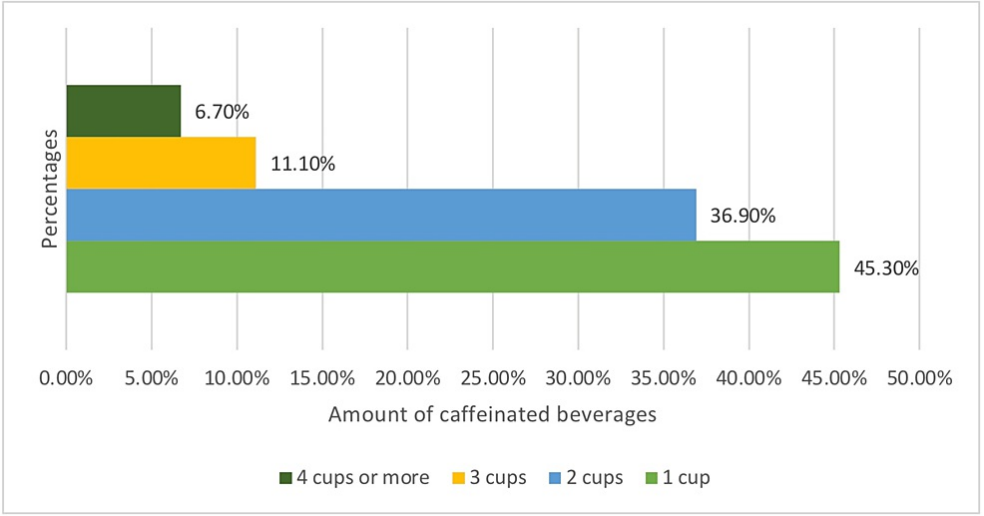
Frequency of caffeinated beverages consumption per day among medical students

Figure 1 represents the total academic performance of medical students based on GPA percentage. 280 (47.20%) of medical students had an average GPA of 85%-95%, while 201 (34%) had an average below that. The remaining students, which accounted for the smallest percentage, had an average GPA above 95%. The most popular caffeinated beverage consumed by medical students was coffee, as shown in Figure 2. The rest of the students were distributed among other caffeinated beverages. Figure 3 displays the frequency of consuming caffeinated beverages per day. Most students 268 (45.30%) consumed only one cup per day, while 218 (36.90%) consumed two cups per day.

Association between participants' demographics characteristics and academic performance

Table 2 shows the results of the association between demographics and the academic performance of medical students. In terms of gender, the majority of male participants 64 (10.8%) had GPAs below 85%, whereas the majority of females 132 (22.2%) achieved GPAs within the average range of 85%-95%. In relation to BMI, the majority of individuals classified as obese 21 (3.5%) and extremely obese 18 (3.04%) have GPAs below 85%. Moreover, the majority of students with GPAs below 85% and GPAs ranging from 85% to 95% were from UQU 41 and 103 (6.9% and 17.36%, respectively). Conversely, most students with GPAs above 95% were from King Saud University. Furthermore, a A significant association was discovered between the academic year in medical school and GPA, as indicated by a significant p-value.

**Table 2:** Relationship between demographics and the academic performance of medical students UQU: Umm Al-Qura University N=593

Gender	GPA percentages			P value
	<85%	85%-95%	>95%	
Male	11.6% (n=69)	10.8% (n=64)	4.3% (n=26)	0.006
Female	12.1% (n=72)	22.2% (n=132)	8.7% (n=52)	
Age				
18-20 years old	3.8% (n=23)	8.6% (n=51)	6.9% (n=41)	0.001
21-23 years old	14.3% (n=85)	18.9% (n=112)	5.4% (n=32)	
24-26 years old	5.57% (n=33)	5.57% (n=33)	0.8% (n=5)	
BMI				
<18.5 (underweight)	1.1% (n=7)	4.05% (n=24)	1.69% (n=10)	0.001
18.5-24.9 (normal)	8.6% (n=51)	19.05% (n=113)	5.9% (n=35)	
25.0-29.9 (overweight)	7.4% (n=44)	7.5% (n=45)	2.19% (n=13)	
30.0-34.9 (obese)	3.5% (n=21)	1.3% (n=8)	2.03% (n=12)	
>35 (extremely obese)	3.04% (n=18)	1.01% (n=6)	1.35% (n=8)	
Physical activity				
Sedentary lifestyle (<5,000 steps per day)	10.12% (n=60)	10.9% (n=65)	6.07% (n=36)	0.127
Low active (5,000-7,499 steps per day)	6.58% (n=39)	9.11% (n=54)	3.5% (n=21)	
Somewhat active (7,500-9,999 steps per day)	4.5% (n=27)	8.7% (n=52)	2.3% (n=14)	
Active (>10,000 steps per day)	2.03% (n=12)	2.36% (n=14)	1.18% (n=7)	
Highly active (>12,500 steps per day)	0.51% (n=3)	1.86% (n=11)	0% (n=0)	
Smoking				
Yes	3.37% (n=20)	4.05% (n=24)	0.34% (n=2)	0.025
No	20.41% (n=121)	28.99% (n=172)	12.82% (n=76)	
Total personal income per month				
Less than 1,500 SR	15.67% (n=93)	24.6% (n=146)	9.6% (n=57)	0.15
1,500-2,000 SR	3.3% (n=20)	4.05% (n=24)	2.3% (n=14)	
More than 2,000 SR	4.72% (n=28)	4.3% (n=26)	1.18% (n=7)	
University				
UQU	6.9% (n=41)	17.36% (n=103)	2.87% (n=17)	0.001
King Abdulaziz University	2.03% (n=12)	1.69% (n=10)	1.01% (n=6)	
King Saud University	0.68% (n=4)	1.35% (n=8)	4.55% (n=27)	
Jeddah University	5.23% (n=31)	1.3% (n=8)	0.5% (n=3)	
Ibn Sina University	3.54% (n=21)	2.8% (n=17)	1.3% (n=8)	
Fakeeh College for Medical Science	0.8% (n=5)	0.3% (n=2)	0% (n=0)	
Batterjee Medical College	2.19% (n=13)	3.88% (n=23)	1.35% (n=8)	
Taif University	2.36% (n=14)	4.2% (n=25)	1.5% (n=9)	
Academic year in medical school				
2^nd^ year	3.2% (n=19)	6.7% (n=40)	5.9% (n=35)	0.001
3^rd^ year	5.4% (n=32)	5.5% (n=33)	2.1% (n=13)	
4^th^ year	3.8% (n=23)	7.4% (n=44)	2.03% (n=12)	
5^th^ year	4.2% (n=25)	6.4% (n=38)	2.1% (n=13)	
6^th^ year	3.7% (n=22)	4.5% (n=27)	0.34% (n=2)	
Intern	3.3% (n=20)	2.3% (n=14)	0.51% (n=3)	
Caffeinated beverages that medical students consume				
Coffee	17.7% (n=105)	24.4% (n=145)	9.7% (n=58)	0.742
Tea	3.04% (n=18)	5.7% (n=34)	1.8% (n=11)	
Soft drinks	1.3% (n=8)	0.6% (n=4)	0.6% (n=4)	
Energy drinks	0.6% (n=4)	1.18% (n=7)	0.5% (n=3)	
Chocolate	1% (n=6)	1% (n=6)	0.34% (n=2)	
Frequency of caffeinated beverages consumption				
1 cup per day	9.6% (n=57)	15.3% (n=91)	6.7% (n=40)	0.629
2 cups per day	9.6% (n=57)	12.3% (n=73)	3.88% (n=23)	
3 cups per day	2.5% (n=15)	3.5% (n=21)	1.69% (n=10)	
4 cups or more per day	2.03% (n=12)	1.8% (n=11)	0.8% (n=5)	

Relationship between caffeinated beverages consumption and academic performance

Table 3 shows the logistics regression analysis for examining the relationship between caffeine consumption and academic performance. In this multivariable analysis, considering the GPA of < 85% as the reference GPA category, the factor associated with GPA of > 95% was the age of participants (adjusted odds ratio (aOR): 0.285, CI: 0.176-0.462, p≤0.001). BMI (aOR: 0.548, CI: 0.430-0.689, p≤0.001) was the factor most associated with the GPA category of 85%-95%. 

**Table 3:** Multivariate regression analysis of the effect of caffeine consumption on academic performance OR: Odds ratio

Associated factors	Academic performance				
	Multivariate logistic regression analysis (N=593)				
	<85%	85%-95%		>95%	
		OR (95% CI)	P-value	OR (95% CI)	P-value
Gender	Ref.	1.273 (0.774-2.096)	0.342	1.464 (0.766-2.800)	0.249
Age	Ref.	0.786 (0.547-1.129)	0.192	0.285 (0.176-.462)	<0.001
BMI	Ref.	0.548 (0.430-.689)	<0.001	0.875 (0.659-1.162)	0.357
University	Ref.	0.932 (0.851-1020)	0.127	0.998 (0.894-1.115)	0.979
Frequency of caffeinated beverages consumption					
1 cup per day	Ref.	1.686 (0.653-4.352)	0.280	1.595 (0.486-5.241)	0.441
2 cups per day	Ref.	1.417 (0.550-3.651)	0.470	0.941 (0.280-3.165)	0.922
3 cups per day	Ref.	1.868 (0.610-5.723)	0.274	1.494 (0.371-6.011)	0.572
4 cups or more per day	Ref.	-	-	-	-

Medical students' perception regarding caffeinated beverages consumption

Table 4 presents the perceptions of medical students regarding the consumption of caffeinated beverages. A notable percentage of students 388 (65.6%) believe that caffeine intake enhances academic performance, while a majority 428 (72.2%) do not think it increases intelligence. Additionally, a significant portion 392 (66.3%) of students feel that caffeine does not boost self-confidence, and a majority 335 (56.5%) do not believe it improves reading speed. However, a significant number 451 (76.1%) of students perceive that caffeine increases study hours, 361 (60.9%) feel it reduces fatigue, and 387 (65.4%) believe it enhances their thinking abilities. Furthermore, a majority 406 (68.5%) do not think caffeine has a negative impact on their mood. On the other hand, a considerable proportion 371 (62.7%) of students believe that caffeine diminishes the quality of their sleep. Interestingly, most students do not believe that caffeinated drinks provoke anger.

**Table 4:** Perception of medical students regarding the consumption of caffeinated beverages

Variables	n	%
Do you feel that caffeine consumption increases academic performance?		
Yes	389	65.6%
No	204	34.4%
Do you feel that caffeine consumption increases intelligence quotient (IQ)?		
Yes	165	27.8%
No	428	72.2%
Do you feel that caffeine increases self confidence?		
Yes	200	33.7%
No	393	66.3%
Do you feel that caffeine increases reading speed?		
Yes	258	43.5%
No	335	56.5%
Do you feel that caffeine increases study hours?		
Yes	451	76.1%
No	142	23.9%
Do you feel that caffeine reduces fatigue?		
Yes	361	60.9%
No	232	39.1%
Do you feel that caffeine improves your ability to think?		
Yes	388	34.6%
No	205	34.6%
Do you feel that caffeine affects your mood negatively?		
Yes	187	31.5%
No	406	68.5%
Does caffeine reduces your sleep quality?		
Yes	372	62.7%
No	221	37.3
Do you feel that caffeinated drinks make you angry?		
Yes	170	28.7%
No	423	71.3%

## Discussion

The marketing of caffeinated beverages has increased, especially towards young adults, and the marketing may be more appealing to college students, particularly athletes. Previous studies have found that caffeinated beverage consumption is a popular practice among medical college students, particularly if the student had insufficient sleep or needed more energy in general while studying for exams, working on major course projects or driving an automobile for a long period of time. Improvement in mental functioning is of interest among college students. We completed this study to determine the frequency of caffeine consumption and its effect on academic performance among medical students in Makkah, Saudi Arabia.

The majority of participants in this research were female (62.2%), which was similar to a United States study conducted among colleges students [10]. Most of the medical students who participated had an average GPA of 85%-95% (47.20%). Additionally, majority of medical students consumed only one cup of caffeinated beverage per day (45.30%), which is consistent with the findings of the aforementioned US study [10].

Regarding the most popular caffeinated beverages, our results demonstrated that most medical students consume coffee more than the other caffeinated beverages (72.30%). This is in agreement with the US study, as most of their students consume coffee too (72.0%) [10]. However, it is inconsistent with the results of an Australian study, which reported that energy drinks were the most popular beverage choice (48%) [9]. Moreover, another study done among university students in Beirut, Lebanon, also reported that energy drinks were the most popular caffeinated beverages among their students [4]. 

We found an association between medical students’ academic performance and gender, as higher academic achievement was found among females with a significant p-value (P=0.006). Moreover, as the age and academic year of medical students increase, their academic performance became higher, as represented by their GPA (P<0.001). Moreover, medical students with a normal BMI have higher achievement (P<0.001). Regarding the relationship between caffeinated beverage consumption and academic performance, our results reported no association. These findings are not in agreement with the Lebanon study, as the authors suggested that energy-drink consumption is associated with lower academic achievement [4]. This discrepancy may arise due to a variation in the preferred caffeinated beverages among our students and their students, leading to disagreement, as energy drink consumption in our study is limited only to (3.70%) of our medical students. 

All types of caffeinated beverages, such as coffee, tea, soft drinks, energy drinks, and chocolate, are easily affordable and available with no restrictions regarding purchase or use. Therefore, primary prevention of excessive caffeine consumption may be beneficial for preventing increased prevalence of energy drinks, which may negatively contribute to the academic achievement of medical students. We suggest adding an educational lecture supported with research to educate medical students. We also recommend further work to explore how these associations hold or differ across all universities in all regions of Saudi Arabia.

Strength and limitations 

This study is the first of its kind in Saudi Arabia to investigate behavior of medical students regarding caffeinated beverages. Furthermore, using two methods in SPSS statistical analysis to explore the association between caffeinated beverage consumption and academic achievement of medical students is a strength for our study. One limitation of this study is that students self-selected to participate and, therefore, higher-achieving students may have been more likely to complete the questionnaire. 

## Conclusions

Caffeinated beverages are popular among Makkah medical college students, with coffee as the most popular choice and energy drinks as the least popular. Most students consume only one cup per day. This behavior contributes to a lack of association between caffeinated beverages consumption and academic achievement. However, as energy drinks are easily affordable and available, primary prevention of excessive caffeine consumption is necessary.

## References

[1] Alabbad MH, AlMussalam MZ, AlMusalmi AM, Alealiwi MM, Alresasy AI, Alyaseen HN, Badar A (2019). Determinants of energy drinks consumption among the students of a Saudi University. J Family Community Med.

[2] Alfaifi MH, Gosadi IM, Alfaifi SM (2022). Assessment of caffeine consumption behavior among Jazan University students in the south of Saudi Arabia: a cross-sectional study. Medicine (Baltimore).

[3] Champlin SE, Pasch KE, Perry CL (2016). Is the consumption of energy drinks associated with academic achievement among college students?. J Prim Prev.

[4] Ghozayel M, Ghaddar A, Farhat G (2015). Energy drink consumption: beneficial and adverse health effects. Int J Health Sci (Qassim).

[5] Health Health, C. (n.d (2024). What that energy drink can do to your body. https://edition.cnn.com/2017/04/26/health/energy-drinks-health-concerns-explainer/index.html.

[6] (2024). Are energy drinks good or bad for you?. https://www.healthline.com/nutrition/energy-drinks.

[7] Mahoney CR, Giles GE, Marriott BP, Judelson DA, Glickman EL, Geiselman PJ, Lieberman HR (2019). Intake of caffeine from all sources and reasons for use by college students. Clin Nutr.

[8] Malinauskas BM, Aeby VG, Overton RF, Carpenter-Aeby T, Barber-Heidal K (2007). A survey of energy drink consumption patterns among college students. Nutr J.

[9] Narayanan A, Gill M, Leem C (2021). Students' use of caffeine, alcohol, dietary supplements, and illegal substances for improving academic performance in a New Zealand university. Health Psychol Behav Med.

[10] Nawrot P, Jordan S, Eastwood J, Rotstein J, Hugenholtz A, Feeley M (2003). Effects of caffeine on human health. Food Addit Contam.

